# Domestic Violence Against Married Women During the COVID-19 Quarantine in Saudi Arabia

**DOI:** 10.7759/cureus.15231

**Published:** 2021-05-25

**Authors:** Fares F Alharbi, Meshal A Alkheraiji, Abdullah A Aljumah, Majid Al-Eissa, Salman S Qasim, Meshal K Alaqeel

**Affiliations:** 1 Mental Health, Ministry of the National Guard Health Affairs, Riyadh, SAU; 2 Research Office, King Abdullah International Medical Research Center, Riyadh, SAU; 3 College of Medicine, King Saud Bin Abdulaziz University for Health Sciences, Riyadh, SAU; 4 Psychiatry, King Saud University, Riyadh, SAU; 5 Psychiatry, King Saud University Medical City, Riyadh, SAU; 6 Pediatrics, Ministry of the National Guard Health Affairs, Riyadh, SAU

**Keywords:** covid-19, domestic violence, married women, abuse, saudi arabia

## Abstract

Background

Before the coronavirus disease 2019 (COVID-19) pandemic, cases of domestic abuse and aggressive behaviour between Saudi married couples were increasing annually, a topic of growing concern both socially and medically. With the forced indoor confinement enacted as a containment measure, international studies regarding domestic abuse indicated an almost unanimous increase in prevalence. This cross-sectional national study aimed to assess the change between the pre-and intra-pandemic prevalence of abuse in Saudi Arabia.

Material and methods

Anonymous data were gathered using a web-based Arabic version of the World Health Organization (WHO) multi-country instrument measuring violence against women residing in Saudi Arabia. The previously validated questionnaire included a series of multiple-choice questions related to demographic information, family infrastructure, experienced situations of abuse, and the severity and form of abuse during the quarantine period, from March 23, 2020, to June 21, 2020. Associations were tested using a two-tailed Pearson's Chi-square test and odds ratios. A binary multivariate logistic regression was used to identify the independent factors associated with domestic violence.

Results

In total, 2254 participants were included in the present study. The majority (n=2129, 94.7%) were Saudi nationals. The highest proportion (n=1022, 45.3%) was in the 30 to 40 years age group. The self-reported prevalence of domestic violence before COVID-19 pandemic and quarantine was 25.4% and 16.6% during the confinement, indicating an overall decrease of 8.8% in the reported cases. Regarding the type of violence, of the 315 (16.6%) women who endured violence since the confinement, the majority (n=301, 95.6%) experienced multiple forms of violent abuse, 264 (87.7%) suffered from psychological/emotional violence, 114 (37.9%) from physical violence, and 50 (16.6%) from sexual violence. Of the group who experienced multiple forms of violence, 120 (39.9%) reported an increase in the frequency and perceived intensity of the violence since the confinement. The only variable that directly increased the likelihood of suffering domestic violence had more than three children [OR = 1.59, P = 0.018].

Conclusions

Contrary to trends observed in other countries, the national prevalence of abusive conduct towards married women showed a marked decrease during the quarantine period-more children directly correlated with a higher reported frequency of being abused. Further studies in neighbouring countries with comparable societies and structures must be conducted to assess the validity of our findings in the context of the global trends of violence in the marital home.

## Introduction

During the coronavirus disease 2019 (COVID-19) pandemic, healthcare services could not meet the exponential increase in demands on resources, leading to the entire population's subsequent forced confinement in almost every affected country, including Saudi Arabia [1-5]. In addition to the forced indoor confinement, other measures implemented and still actively enforced include the compulsory wearing of surgical masks or visors in enclosed public spaces, mandatory self-isolation with suspected infection, and physical distancing and isolation [6-8]. The social and community health consequences of these measures are now becoming more apparent and concerning as the rate of depression, suicide, substance abuse, and domestic violence are steadily increasing [9-11]. As a general definition, domestic violence is any purposeful attack by one or more close relatives or acquaintances through psychological, physical, or sexual acts of aggression or violence with an intent to compel, control, or debase the victim [10,12,13].

In a world where a person is legally bound to remain indoors, domestic violence may potentially have the most lasting and far-reaching harm. Unlike most other consequences of forced confinement, domestic violence endangers the individual and adversely affects all those around to varying degrees. The consequences often present in later life as post-traumatic stress disorder or academic failure [12-14]. Literature related to this subject offers substantial support for these practices' serious nature, providing evidence that between a quarter and two-thirds of the perpetrators are intimate partner. Approximately one-third of the cases require urgent medical assistance. Literature also reports problems related to academic performance in not attending classes or altogether dropping out from the school of children who suffered domestic violence [15-17].

In Saudi Arabia, studies before the pandemic and enforced quarantine reported that the lifetime prevalence of domestic violence ranged between 33% and 45% for married women [13,18,19], high in the context of a comparatively calm period. The first step to controlling such a problem is to assess its current state. The current study aimed to evaluate the existing prevalence of domestic violence in Saudi families and establish a predicted change rate using past and present values as reference. In addition, the study aimed to determine whether factors related to the demographic information, financial status or matrimony could identify individuals who are at a higher risk of domestic abuse.

## Materials and methods

This was a cross-sectional study using a convenience sampling technique. The inclusion criteria were married women, at least 18 years of age, living in Saudi Arabia during the quarantine period, from March 23, 2020, to June 21, 2020. Eligible participants were approached through social media, including but not limited to Twitter and WhatsApp. The data were collected using an Arabic version of the World Health Organization (WHO) multi-country instrument related to violence against women [18,20]. The self-assessment questionnaire included sections about the demographic characteristics of the married women, such as age, level of education, income, and occupation. The questionnaire also covered the type of consanguinity between married women and their spouses. The duration of the marriage, state of living with another wife in the same house, and the presence of children from another wife were also assessed. In addition, the questionnaire included questions about the risks and protective factors of intimate partner violence in the four domains of domestic abuse against women: physical, psychological, sexual, and economic abuse. In each domain, data were collected as present and past exposure to violence and frequency of violence (always, sometimes, rarely, or never). Screening for violence was considered positive when responding to a question about intimate partner violence was (always). The prevalence of the different forms of domestic violence was estimated based on the number of responses. The prevalence of violence in Saudi Arabia was estimated based on the positive responses to any form of violence towards the married female. The sections related to general health were not included in the current study for the sake of simplicity and time constraints. 

The sample included both Saudi and non-Saudi married women. According to the Saudi General Authority for Statistics, 4,832,830 Saudi and non-Saudi females are married between the age of 15 and 64 years [21]. According to the Raosoft Digital Sample Size Calculator (Raosoft, Inc., Seattle, WA; http://www.raosoft.com/samplesize.html), the minimum recommended sample size get a representative randomized sample with a 99% level of confidence, and a 5% margin of error was approximately 700 married women. The data were compiled in Microsoft Excel 2010 (Microsoft, Redmond, WA, USA) and analyzed through the Statistical Package for Social Sciences (SPSS) Statistics version 23 (IBM Corp., Armonk, NY, USA). Categorical variables are represented as frequency and percentage, and associations were tested using a two-tailed Pearson's Chi-square test and odds ratios. A binary multivariate logistic regression analysis was used to identify the independent factors associated with domestic violence. A p-value of ≤ 0.01 for the associations between the variables and a p-value of ≤ 0.05 for the odds ratio precision testing was deemed significant. 

The Institutional Review Board approved the present study of King Abdullah International Medical Research Center, Ministry of National Guard-Health Affairs, Riyadh, Saudi Arabia (approval number RC20/218/R, April 28, 2020). Patient data were obtained and used by only the study team, ensuring patient confidentiality and privacy.

## Results

In total, 2254 participants were included in the present study. The majority (n=2129, 94.7%) were Saudi nationals. The highest proportion (n=1022, 45.3%) was in the 30 to 40 years age group, 1408 (65.7%) had a college-level education, and a half (n=1079, 50.2%) were employed. Of the employed group, 688 (72.3%) were government employees. A summary of the demographic characteristics of the participants is shown in Table 1.

**Table 1:** Demographic characteristics of married women

Characteristic	n (%)
Nationality (n=2247)	
Saudi	2129 (94.7)
Non-Saudi	118 (5.3)
Age group, years (n=2254)	
30 and below	702 (31.1)
31-40	1022 (45.3)
41-50	387 (17.2)
Above 50	143 (6.3)
Number of children (n=2254)	
None	244 (10.8)
1-2	724 (32.1)
3-5	794 (35.2)
More than 5	492 (21.8)
Level of education (n=2144)	
Able to read and write	13 (0.6)
Below high school	64 (3.0)
High school	294 (13.7)
College	1408 (65.7)
Post-graduate studies	365 (17.0)
Employment (n=2151)	
Employed	1079 (50.2)
Unemployed	1072 (49.8)
Type of employment (n=951)	
Government employee	688 (72.3)
Private sector employee	200 (21.0)
Others	63 (6.6)

The self-reported prevalence of domestic violence before the COVID-19 pandemic and quarantine was 25.4% and 16.6% during confinement, indicating an overall decrease of 8.8% in the reported cases. Regarding the type of violence, of the 315 (16.6%) women who endured violence since the confinement, the majority (n=301, 95.6%) experienced multiple forms of violent abuse, 264 (87.7%) suffered from psychological/emotional violence, 114 (37.9%) from physical violence, and 50 (16.6%) from sexual violence. Of the group who experienced multiple forms of violence, 120 (39.9%) reported an increase in the frequency and perceived intensity of the violence since the confinement, 128 (42.5%) reported an unchanged frequency and intensity of violence. The remaining 53 (17.6%) noted a reduction in violent outbursts (Table 2).

**Table 2:** Prevalence, type, and intensity of domestic violence among married women COVID-19: coronavirus disease 2019 *No response for outcome variable (n=353)

Outcome variables	n (%)
Prevalence of domestic violence (n=1901)*	
Before COVID-19	483 (25.4)
Since COVID-19	315 (16.6)
Did not encounter domestic violence	1103 (58)
Type of domestic violence (multiple responses) (n=301)	
Physical	114 (37.9)
Psychological/emotional	264 (87.7)
Sexual	50 (16.6)
Frequency and intensity of domestic violence since the COVID-19 pandemic (n=301)	
Increased	120 (39.9)
Unchanged	128 (42.5)
Decreased	40 (13.3)
Stopped	13 (4.3)

Of all of the variables, only the number of offspring had a statistically measurable effect on the probability of being subjected to violence by the husband or close family members. The results indicated that with more than three children, the probability of abuse increased proportionally with each successive child [OR = 1.59, P = 0.018] (Table 3).

**Table 3:** Association between prevalence of domestic violence and sociodemographic characteristics of married women CI: confidence interval; OR: odds ratio

Characteristic	Domestic violence		P-value	OR (CI 95%)
	Yes	No		
Nationality (n=482; 1415)				
Saudi	452 (25.1)	1352 (74.9)	0.120	1.0 (reference)
Non-Saudi	30 (32.3)	63 (67.7)		1.42 (0.91-2.30)
Age group, years (n=483; 1418)				
30 and below	129 (22.2)	453 (77.8)	0.188	1.0 (reference)
31-40	232 (26.9)	629 (73.1)		1.29 (1.01-1.66)
41-50	93 (27.0)	251 (73.0)		1.19 (0.75-1.91)
Above 50	29 (25.4)	85 (74.6)		
Vital status of the father (n=481; 1416)				
Alive	416 (24.7)	1267 (75.3)	0.258	1.0 (reference)
Deceased	65 (30.5)	148 (69.5)		1.14 (0.91-1.42)
Vital status of the mother (n=481; 1415)				
Alive	416 (24.7)	1267 (75.3)	0.067	1.0 (reference)
Deceased	65 (30.5)	148 (69.5)		1.34 (0.98-1.83)
Number of male siblings (n=483; 1418)				
None	20 (25.0)	60 (75.0)	0.359	1.0 (reference)
1-5	410 (26.0)	1167 (74.0)		1.05 (0.63-1.77)
More than 5	53 (21.7)	191 (78.3)		0.83 (0.46-1.50)
Number of female siblings (n=483; 1418)				
None	26 (32.9)	53 (67.1)	0.258	1.0 (reference)
1-5	367 (24.8)	1111 (75.2)		0.67 (0.41-1.09)
More than 5	90 (26.2)	254 (73.8)		0.72 (0.43-1.22)
Number of children (n=483; 1418)				
None	45 (19.3)	188 (80.7)	0.018	1.0 (reference)
1-2	166 (23.7)	535 (76.3)		1.29 (0.89-1.87
3-5	212 (27.6)	557 (72.4)		1.59 (1.11-2.28)
More than 5	60 (30.3)	138 (69.7)		1.82 (1.16-2.83)
Level of education (n=483; 1415)				
Able to read and write	2 (25.0)	6 (75.0)	0.267	1.22 (0.24-6.18)
Below high school	19 (33.9)	37 (66.1)		1.88 (1.02-3.47)
High school	67 (26.5)	186 (73.5)		1.32 (0.90-1.93)
College	324 (25.9)	926 (74.1)		1.28 (0.96-1.71)
Post-graduate studies	71 (21.5)	260 (78.5)		1.0 (reference)
Employment (n=483; 1418)				
Employed	226 (24.0)	717 (76.0)	0.152	1.0 (reference)
Unemployed	257 (26.8)	701 (73.2)		1.16 (0.95-1.43)
Type of employment (n=213; 650)				
Government employee	161 (25.9)	461 (74.1)	0.320	1.61 (0.79-3.26)
Private sector employee	42 (22.7)	143 (77.3)		1.35 (0.63-2.90)
Others	10 (17.9)	46 (82.1)		1.0 (reference)

## Discussion

Before the COVID-19 pandemic, studies in Saudi Arabia indicated a high prevalence of domestic violence and abuse [13,18,19,22,23]. After the pandemic containment measures were implemented, studies reported that the rates were increasing in most countries and communities, mandating additional measures to control and report domestic violence [10,14]. The same trend was expected when we planned this intra-pandemic study in the Saudi population. However, we found that the national prevalence of domestic violence decreased by 8.8% during the quarantine period. This result, while encouraging, still requires an explanation as little corroboration is available in almost all literature related to the subject during the pandemic.

It should be noted that the current study measured domestic violence during the COVID-19 pandemic and not the lifetime risk of domestic violence. For example, if a woman was not subjected to domestic violence during the quarantine, this does not necessarily mean that she did not encounter domestic violence before the quarantine. The findings should not be incorrectly applied to a lifetime prevalence of domestic violence. In other communities, domestic violence during the COVID-19 pandemic showed different prevalence rates. Studies conducted in the United States of America reported a 10 to 27% increase in domestic violence during the COVID-19 pandemic [24,25]. In the WHO Europe member states, there was a 60% rise in emergency calls from women subjected to violence by their intimate partner [26]. This disparity could be explained if it is taken into account that culturally distinct groups have varying definitions and valuations of domestic violence boundaries. Another possible explanation is that the forced isolation between family members may have had a positive impact on intra-family relations by reducing the number of people per house at any given time. In Saudi society, it is prevalent for the extended family of the wife or husband to congregate in one abode, living in very close proximity with each other, affording little privacy, space, or peace of mind for the large part of the day and night. During the quarantine, however, extended family members were required to stay at home with their immediate family only, resulting in less overcrowding, more attention and intimacy between the husband and wife, and fewer outbursts of dissatisfaction by either party. The uncertainty that shrouds all societies' future due to the quarantine and pandemic may have forced families and couples to become more lenient and understanding, which is essential for cohabitation when no other options are available. Practicality and necessity are strong motivators for dialogue. 

There were several limitations to the present study. Only those who are literate and had access to the internet, whether through smartphones or laptops, could complete the questionnaire. Considering the quarantine period, however, this was the most effective way of reaching out to people to recognize domestic violence among married women. Moreover, the spread of participants across Saudi Arabia may have been limited to individuals with an interest in and experience of domestic abuse, limiting the generalizability of our findings. Although the incidence of violence during the quarantine was lower than in other studies, the victims' perceptions of the intensity of violence were noticeably higher than before the quarantine. The necessary tools and methods to quantify this perceived increase of the intensity of violence were not part of this research design and authorized scope. As records of past events related to domestic violence are not available, we cannot offer any view. In addition to the fear or uncertainty of being discovered by the abusive spouse, there is also a robust socially imposed taboo on speaking openly of what goes on between couples or close family behind closed doors, and that for this reason, the number of reports may not be a true representation of actuality [10,14,24].

## Conclusions

During the quarantine period, the prevalence rate of domestic violence in Saudi Arabia showed a marked decrease compared to the rates reported before the COVID-19 pandemic. The current study outcomes are inconsistent with the literature, allowing a cautiously optimistic outlook for the future of our society and women's rights. We endorse more measures and strategies to provide assistance and support for female victims of domestic violence. Awareness campaigns should be implemented in Family Medicine and pre-marital examination clinics, highlighting the impact of domestic violence on interpersonal relationships. Additional studies in neighbouring countries with comparable societies and structures must be conducted to assess the validity of our findings in the context of the global trends in violence in the marital home.

## Appendices

**Table 4:** Domestic Violence Questionnaire

Domestic Violence Questionnaire	
Nationality	☐ Saudi ☐ Non-Saudi
Age	
Is your father alive?	☐ Yes ☐ No
Is your mother alive?	☐ Yes ☐ No
How many male siblings do you have?	☐ None ☐ 1-5 ☐ More than 5
How many female siblings do you have?	☐ None ☐ 1-5 ☐ More than 5
How many children do you have?	☐ None ☐ 1-2 ☐ 3-5 ☐ More than 5
What is the highest level of education that you achieved?	☐ Able to read and write ☐ Below high school ☐ High school ☐ College ☐ Post-graduate studies
Do you have a job or private source of income?	☐ Yes ☐ No
If yes, what is the nature of the job?	☐ Government employee ☐ Private sector employee ☐ Others
Before the COVID-19 pandemic, were you ever exposed to marital violence?	☐ Yes ☐ No
Since the COVID-19 pandemic, were you ever exposed to marital violence?	☐ Yes ☐ No
Since the COVID-19 pandemic, has the frequency of violence (times of its occurrence) and intensity changed?	☐ Increased ☐ Continued the same ☐ Decreased ☐ Stopped
What is the nature of the abuse? (you can choose more than one answer)	☐ Physical ☐ Psychological/Emotional ☐ Sexual
